# Regular Deployment of Wireless Sensors to Achieve Connectivity and Information Coverage

**DOI:** 10.3390/s16081270

**Published:** 2016-08-12

**Authors:** Wei Cheng, Yong Li, Yi Jiang, Xipeng Yin

**Affiliations:** School of Electronics and Information, Northwestern Polytechnical University, Xi’an 710072, Shaanxi, China; ruikel@nwpu.edu.cn (Y.L.); jiangyiv88@nwpu.edu.cn (Y.J.); yinxipeng@nwpu.edu.cn (X.Y.)

**Keywords:** information coverage, connectivity, WSNs

## Abstract

Coverage and connectivity are two of the most critical research subjects in WSNs, while regular deterministic deployment is an important deployment strategy and results in some pattern-based lattice WSNs. Some studies of optimal regular deployment for generic values of *r_c_*/*r_s_* were shown recently. However, most of these deployments are subject to a disk sensing model, and cannot take advantage of data fusion. Meanwhile some other studies adapt detection techniques and data fusion to sensing coverage to enhance the deployment scheme. In this paper, we provide some results on optimal regular deployment patterns to achieve information coverage and connectivity as a variety of *r_c_*/*r_s_*, which are all based on data fusion by sensor collaboration, and propose a novel data fusion strategy for deployment patterns. At first the relation between variety of *r_c_*/*r_s_* and density of sensors needed to achieve information coverage and connectivity is derived in closed form for regular pattern-based lattice WSNs. Then a dual triangular pattern deployment based on our novel data fusion strategy is proposed, which can utilize collaborative data fusion more efficiently. The strip-based deployment is also extended to a new pattern to achieve information coverage and connectivity, and its characteristics are deduced in closed form. Some discussions and simulations are given to show the efficiency of all deployment patterns, including previous patterns and the proposed patterns, to help developers make more impactful WSN deployment decisions.

## 1. Introduction

Two of the most critical research issues in wireless sensor networks are achieving full area coverage and maintaining the connectivity of the whole network [1,2,3]. In a WSN, each sensor node has a limited sensing range *r_s_*, and a limited communication range *r_c_*. The collection of the sensing range of all sensor nodes is regarded as the sensing coverage of whole network [2], which implies how well the area of environment is sensed. In addition, keeping the WSN connected is also important because sensing data may need to be sent to the data center. [3]. Each sensor is assumed to have limited communication range *r_c_*, which usually may be different from the sensing range *r_s_*. For instance, the communication range of an Extreme Scale Mote (XSM) platform is about 30 m, while the sensing range of the acoustics sensor for vehicle detection is about 55 m [4].

According to the accessibility of the monitored area, there are two sensor deployment strategies: deterministic sensor deployment and random sensor deployment. In general, pattern-based lattice WSNs resulting from deterministic sensor deployment provide better coverage and connectivity, compared to random deployments [5]. On the problem of achieving both coverage and connectivity for sensor deployment, some results are available in past research: when the communication range *r_c_* is at least twice the sensing range *r_s_*, then covering an area implies a full connectivity in WSN [6]. If *r_c_* ≥$\sqrt{3}r_{s}$, then a regular triangle deployment can achieve full coverage and connectivity, and is the most efficient in terms of the density of sensors needed. When *r_c_* = *r_s_*, the strip-based deployment is near optimal [7]. However, few results are known for generic values of *r_c_*/*r_s_* [8], since in practice it can take any value. Consequently, it is important to find the optimal deployment pattern to obtain better coverage and connectivity for generic values of the ratio of communication range and the sensing range, i.e., *r_c_*/*r_s_*.

On the other hand, the coverage issue usually depends on the sensing model of sensor nodes in WSNs. Two sensing models have been studied in the past research. One is when a sensor could cover a region that is a disk centered at the sensor node, with a radius equal to its sensing range [1,2,3]. In this sensing model, a specific point is deemed to be covered if it is within the sensing disk of any sensor node. This definition of coverage model is regarded as physical coverage. However, the other type of sensing model is where the sensing accuracy is dependent on the distance between the specific point and the sensor node [9]. Since the sensing intensity from a sensor to a specific point can be regarded as a function of their distance, each specific point of the field can be usually determined by a sensing intensity, which can be measured by its close-by sensor nodes. Every point in the whole region could be deemed to be covered based on this sensing model with different level of sensing intensities. These observations could reform the definition of coverage model in WSNs, thus the new type of information coverage based on distributed estimation theory was proposed by Wang in [9,10,11]. Based on different event scenarios and detection techniques, many detection coverage models have also been proposed in past studies [12,13,14,15,16]. In [17], Xiang focused on detection issues by using a probabilistic sensing model with five different collaborative detectors based on spatial correlation and signal detection theory, and proposed a scheme of signal detection and coverage that completes the seamless 3D space coverage. In [18], Naranjo proposed a new technique to organize the advanced nodes and to select the CHs in WSNs. which can take advantage of data fusion.

In deterministic deployments, there are another type of works which are heterogeneous deployments. In [19], Zalyubovskiy studied the problem of energy-efficient area coverage by the regular placement of sensors with adjustable sensing and communication ranges, and proposed new density control models that considerably improve coverage using sensors with two sensing ranges. In [20], Abbas examined the complete coverage problem when circular disks of two different radii are used, and showed that more efficient coverage can be obtained through configurations of heterogeneous disks as compared to the homogeneous case. There are many extended studies about k-coverage issue for WSNs. Ahmadi [21] reviewed efficient routing algorithms for preserving k-coverage in a sensor network and then proposes an effective technique for preserving k-coverage and the reliability. Li [22] explored the deployment efficiency with regular topology patterns under such cases as full sensing coverage, k-coverage and l-connectivity. Kim [23] presented how to form the regular deployment patterns to achieve p-coverage and q-connectivity. Birjandi [24] compared some regular sensor deployments based on the required sensor density to k-cover sensing area.

Although coverage and connectivity have been studied in recent studies in both physical or probabilistic coverage [25,26,27,28,29,30,31] and coverage by data fusion [32,33,34], the value of *r_c_*/*r_s_* which they considered are fixed mostly. On the issue of optimal regular deployment for generic values of *r_c_*/*r_s_*, some studies are shown recently [1,8]. However, these results are mostly restricted to disk sensing model. Meanwhile *information coverage* is proposed based on data fusion of sensors, and the deterministic deployment under information coverage is studied in [9], while the random deployment under this model is studied in [10]. Nevertheless, the optimal deployment to achieve both full coverage and full connectivity under information coverage model, for generic values of *r_c_*/*r_s_*, has not been completely studied. In response to the above, in this paper, we study optimal deployment patterns for full information coverage and full connectivity in WSNs, for generic values of *r_c_*/*r_s_*, and then try to design some novel data fusion strategies for deployment patterns.

We summarize recent related studies in Table 1. From this table we can find that there are many studies focused on connectivity and coverage issues for WSNs, however there are still some open problems to be resolved. This paper is motivated by the following questions:

(1) Intuitively, information coverage can take advantage of the collaboration of sensors effectively to decrease the number of sensors needed, as in some past studies. However, when we consider coverage and connectivity, the generic values of *r_c_*/*r_s_* could affect the superiority of regular deployment in information coverage. Hence, we intend to answer the question: how good are some regular deployment patterns in information coverage, when the value of *r_c_*/*r_s_* is varied.

(2) Moreover, since the superiority of information coverage comes from the data fusion of the collaborative sensors, we try to find some new strategy of data fusion on sensors cooperation to further enhance the sensor deployment in information coverage.

The main contributions of this paper are as follows:

(1) For regular pattern-based lattice WSNs, the relation between variety of *r_c_*/*r_s_* and density of sensors needed to achieve information coverage and connectivity is deduced in closed form, rather than from known results under the physical coverage model used in past studies.
(2) A dual triangular pattern deployment based on a novel data fusion strategy is proposed, which could utilize collaborative data fusion more efficiently.
(3) The strip-based deployment is extended to a new pattern to achieve information coverage and connectivity, and the results of its characteristic are deduced in closed form.

Finally, some simulations are carried out to show the efficiency of all deployment patterns, including previous patterns and the proposed patterns, to thus help developers make more impactful decisions on the deployment of WSNs.

The organization of this paper is as follows: Section 2 gives some preliminary information about coverage models. In Section 3, regular pattern-based deployments are analyzed, including both physical and information coverage-based schemes, and a new data fusion strategy in regular pattern- based deployments is proposed. Section 4 extends the strip-based deployment from physical coverage to information coverage. Section 5 provides some discussion and extensions of our work, then we evaluate the performance of deployment patterns by numeric simulations in Section 6. Finally, Section 7 is the conclusion.

## 2. Preliminary Information

### 2.1. Physical Coverage

The simple disk/Boolean sensing model has been widely used in numerous studies of WSNs [1,2,3,4,5,6,7,8,12,13,14]. In the disk/Boolean sensing model, each sensor node has a determinate sensing range *r_s_*. A particular sensor node could only sense the phenomenon within its sensing range. Under this sensing model, a point is said to be “physically covered” by a sensor if the Euclidean distance between the point and the sensor is no more than the sensing range *r_s_*. The disk/Boolean sensing model is proverbially adopted for its simplicity and convenience for theoretical analysis. Mathematically, the probability that a point *p* is covered by a sensor node *s_i_* can be expressed as:

(1) $$\text{Pr}\{p\in D(s_{i})\}=\left\{ \begin{array}{ll} 1, & d(p,s_{i})\leq r_{s}\\ 0, & d(p,s_{i})>r_{s} \end{array}\right.$$

where *d*(*p,s_i_*) is the Euclidean distance between point *p* and the sensor node *s_i_*.

### 2.2. Information Coverage

**Figure 1:**
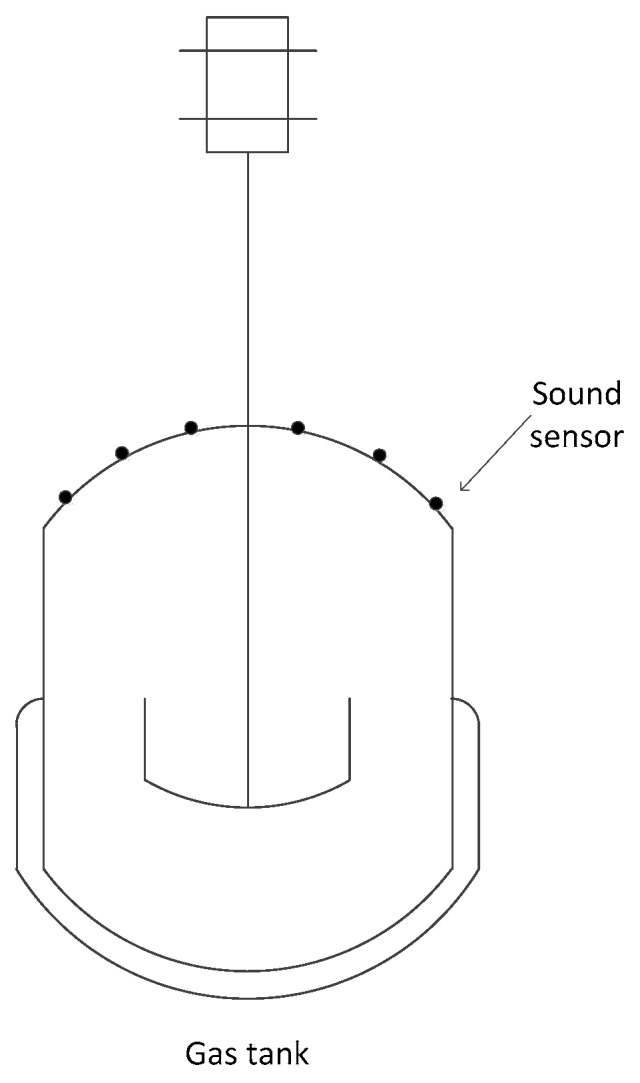
A WSN for monitoring gas leakage.

An important application of sensor networks is to detect features of events/targets within the sensor field. The detection probability of a space point by a sensor is usually related to the distance between them. However, the detection probability of a space point relative to a set of sensors is no longer simply computed as the addition of the detection probability of the point relative to each single sensor. Instead, *data fusion* can be used to derive the detection probability for different event scenarios [11]. Consider a specific application scenario, where wireless acoustic sensors are deployed to measure the sound intensity caused by leakage of a gas tank [10], as shown in Figure 1. If leakage of gas happens, the intensity of the sound could be measured by the adjacent acoustic sensors, and then the sensor measurements could be sent to the sink node. Consequently, the sink could process these measurements to deduce the events in the sensing field. More specifically, the sink could carry out parameter estimation from these gathering measurements for a given point to deduce the event of gas leakage. If the estimation of this point is considered to be reliable and close to some prior knowledge of sound intensity while the leakage took place, a leakage event can be deduced. That is to say, if the estimation on a point is regarded as reliable, the point could be deemed to be covered.

As the example stated above we used the information coverage model which is defined in [9,10]: Consider a set of *K* distributed sensors, each sensor knows its locations and acquire a measurement on an unknown parameter *θ* of an event/target at some location and time. Let ${\hat{\theta }}_{K}$ and ${\tilde{\theta }}_{K}={\hat{\theta }}_{K}-\theta$ denote the estimate and the estimation error by *K* sensors, and *d_i_*, *i* = 1,2,…,*K* denote the normalized distance between a sensor *i* and the location with parameter *θ*. The parameter *θ* is assumed to decay with distance. The measurement of the parameter *θ*, at a sensor may be disturbed by an additive noise *n_i_*. Thus $z_{i}=\theta /d_{i}^{\alpha }+n_{i}$, α > 0, and the goal of a parameter estimator is to estimate *θ* by the corrupted measurements *z_i_*. Let $\hat{\theta }$ and $\tilde{\theta }=\hat{\theta }-\theta$ express the parameter estimate and the estimation error, respectively. When *K* measurements are available, some estimators could be used to estimate *θ*, such as best linear unbiased estimator. Assuming that all noises are independent and are Gaussian noises with zero mean and *σ_i_* standard deviation, then the estimate ${\hat{\theta }}_{K}$ based on the best linear unbiased estimator can be given by:

(2) $${\hat{\theta }}_{K}=\frac{\sum_{i=1}^{K}d_{i}^{-\alpha }\sigma_{i}^{-2}z_{i}}{\sum_{i=1}^{K}d_{i}^{-2\alpha }\sigma_{i}^{-2}}$$

and then this estimator could achieve a minimum mean squared error (MSE) on ${\hat{\theta }}_{K}$, which can be expressed as:

(3) $${\tilde{\theta }}_{K}=\frac{\sum_{i=1}^{K}d_{i}^{-\alpha }\sigma_{i}^{-2}n_{i}}{\sum_{i=1}^{K}d_{i}^{-2\alpha }\sigma_{i}^{-2}}$$

If some event that has happened at a particular position could be estimated with a guaranteed estimation error, this position could be considered as being “information covered” by these cooperative sensors. Note that the estimation error ${\hat{\theta }}_{K}$ is a random variable with zero mean and variance $\sigma_{i}^{2}$. The probability that the absolute value of the estimation error will be less than a constant *A* is larger than a given threshold *ε* can be expressed as: $\text{Pr}\left\{\left|{\tilde{\theta }}_{K}\right|\leq A\right\}$ ≥ *ε*, where 0 ≤ *ε* ≤ 1. We could use the probability that the absolute value of the estimation error is less than a given threshold A, to measure how well a point is covered. The larger this probability is, the more reliable the estimate. When it is larger than a predefined threshold *ε*, we can define the information coverage for *K* cooperative sensors. In another word, a point is said to be “information covered” by *K* sensor nodes, that coverage model could be regarded as (*K,ε*)-covered at the point if there exist *K* sensors to estimate a parameter cooperatively so as to achieve $\text{Pr}\left\{\left|{\tilde{\theta }}_{K}\right|\leq A\right\}$ ≥ *ε*, where 0 ≤ *ε* ≤ 1. This definition implies if any parameter on this point could be estimated by *K* sensors so that the probability that the absolute value of the estimation error is not more than a constant *A* is not less than than *ε*. Therefore, an area is said to be completely (*K,ε*)-covered if all the points in this area are (*K,ε*)-covered.

For simplicity, the following assumptions are made: all noises are Gaussian, then the sum of these noises is still Gaussian with zero mean and variance; assuming that all noises have the same variance $\sigma_{i}^{2}=\sigma^{2}$, *k* = 1,2,…,*K* hence there is:

(4) $$\text{Pr}\left\{\left|{\tilde{\theta }}_{K}\right|\leq A\right\}=1-2Q(\frac{A}{\sigma \sqrt{{\left(\sum_{i=1}^{K}d_{i}^{-2\alpha }\right)}^{-1}}})$$

where $Q\left(x\right)=\frac{1}{\sqrt{2}\pi }\int_{x}^{\infty }\text{exp}\left(-\frac{t^{2}}{2}\right)dt$. For simplicity of comparison with the physical coverage, we transform all distance *D_i_* into a normalized distance *d_i_* = *D_i_*/*r_s_*, where *r_s_* is the sensing range in physical coverage model, that is same as the sensing range related distance where the estimation error equals threshold *ε*, thus there is $Q\left(\frac{A}{d_{1}^{\alpha }\sigma }\right)=\frac{1}{2}\left(1-\varepsilon \right)$ implies the (1,*ε*)-covered sensing range. For simplicity, we set *A* = *σ* and *α* = 1, then the sensing range achieves information coverage in a single sensor case, i.e. (1,*ε*)-covered. When *d_i_* = 1, it means that *D_i_* = *r_s_*, and the threshold *ε* can be calculated accordingly, hence *ε* = 0.683. In this paper, we define ${\hat{r}}_{s}$ as the sensing range for (1,*ε*)-coverage, and it can be computed as ${\hat{r}}_{s}=\frac{r_{s}}{Q^{-1}\left(\frac{1-\varepsilon }{2}\right)}$, where *Q*^–1^(*x*) is the inverse of the *Q*-function.

## 3. Regular Pattern Based Deployment

For WSNs, regular pattern based deployment is also named lattice deployment. In this section, we consider three common regular patterns of deployment: hexagon, square and equilateral triangle, that are commonly used in practice for deployment convenience. Then in Section 3.3, we propose a dual triangular pattern under the information coverage model.

### 3.1. Lattice Deployment under Physical Coverage

It is well-known that placing disks on the vertices of some regular lattice (equilateral triangle, square or hexagon), as illustrated in Figure 1, is an efficient approach achieving *full coverage* of a two-dimensional region [1,8]. In lattice deployment of WSNs, sensor nodes are designedly and accurately placed at deterministic positions as some regular patterns. This deployment of regular lattice specially suits for deterministic deployment of WSNs, as its convenience and efficiency. As a result, sensors could cover a plane in succession without any overlapping regions.

Assume that a field of interest is a two-dimensional region with area A. A number of *N* sensors are deployed in this target region A, and the position of sensor *i* can be expressed by (*x_i_,y_i_*) for *i* = 1,2,…,*N* over the two-dimensional region. In a pattern-based deployment of WSN, the sensors’ positions conform to the geographical pattern shapes. First, we provide some definitions about the coverage which is similar as in [8]:

**Definition 1 [Voronoi Polygon, Area Per Node (APN)].**  *Let {p_1_,p_2_,…p_n_} be a set of n points on an Euclidean plane S. The Voronoi polygon V(p_i_) is a polygon whose interior consists of all points in S that are closer to a particular point p_i_ than to any other point. i.e., V(p_i_) := {x∈S:*$\forall j\in \left[1,n\right],d\left(x,p_{i}\right)\leq d\left(x,p_{j}\right)\};$ *Area Per Node (APN) denotes the area of a Voronoi polygon, We could use this to represents the average contribution to the QoS of network, such as coverage and communication, for any sensor node*.

**Figure 2:**
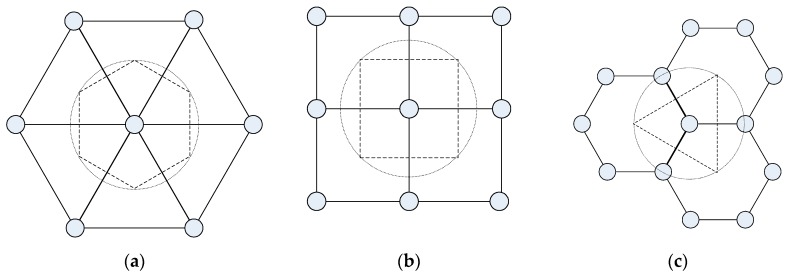
Three popular regular patterns of deployment: (**a**) equilateral triangle; (**b**) square; (**c**) hexagon, and corresponding Voronoi polygons for a sensor node in lattice patterns.

We then present analysis on the coverage and connectivity on regular pattern-based deployment strategy for lattice WSNs, as stated in Figure 2, which is all based on the conventional physical coverage model described in above section. It also illustrates the Voronoi polygon area for a sensor node in lattice deployment with triangular, square and hexagonal pattern, respectively [1,30].

In a lattice deployment, the area of the *APN* (see Definition 1), which is denoted by *γ*, could be expressed as follows [8]:

(5) $$\gamma =\frac{A_{p}}{N_{p}}N_{n}$$

where *A_p_* is the area of the lattice, and there are *N_p_* sensor nodes that constitute a lattice, while each sensor node is share by *N_n_* lattices. For instance, as stated in Figure 1, in a triangular lattice pattern, *N_p_* = 3 and *N_n_* = 6; and in a square pattern, *N_p_* = 4 and *N_n_* = 4 in a hexagon pattern, *N_p_* = 6 and *N_n_* = 3.

Since the concept of *APN* represents the mean contribution of each sensor node to the coverage in a lattice deployment, we could utilize it to evaluate the efficiency of a given pattern. For each lattice pattern, we expect to maximize its *APN*, so as to guarantee that any point in the deployment area is covered by sensor nodes, while the whole network keeps connected.

In the pattern-based lattice WSNs, sensors are precisely placed at desirable positions in a deterministic fashion following regular patterns. As stated in Figure 2, popular patterns including square, equilateral triangle and hexagon that can be repeated to cover a two-dimensional region without leaving any overlapping areas are widely adopted in practice due to the simplicity and the convenience of deployment. We focus on these patterns of regular tiling, which is equivalent to the edge-to-edge tiling by congruent regular polygons. There must be six equilateral triangles, four squares or three regular hexagons at a vertex, yielding the three regular patterns. There are some analyses in [1,8,9] about these patterns, while we would further study their efficiency in providing full information coverage and full connectivity simultaneously under the same context.

It was described in [1] that the area of the Voronoi polygon for a sensor represents the average contribution to the QoS of WSN, such as coverage and connectivity. Therefore it can determine the required number of sensors on the field of interest. To be specific, given an application field with area *S* and *γ_max_* is maximum *APN* of the Voronoi polygon for a sensor in a pattern-based lattice WSNs, the sensor density [29] on the field of interest to achieve both full coverage and full connectivity can be estimated as:

(6) $$\rho_{\text{cov},con}=\frac{1}{\gamma_{\text{max}}}$$

To achieve both full (*K,ε*) coverage and full connectivity in a WSN, each point in the target area should be covered and all sensors should be connected. Let *γ_cov_* and *γ_con_* is the area of the Voronoi polygon for full (*K,ε*) coverage and full connectivity respectively, so the smaller one, of *γ_cov_* and *γ_con_*, is the design bottleneck and determines the maximal area of the Voronoi polygon of a sensor for both full (*K,ε*) coverage and full connectivity, which is similar to the analysis given by [1,8]. Finally the maximum *APN* for actual deployment is determined by *γ_cov_* and *γ_con_*, that is virtually determined by the sensor’s capability of communication range *r_c_* and sensing range *r_s_*:

(7) $$\gamma_{\text{max}}=\text{min}\{\gamma_{\text{cov}},\gamma_{con}\}$$

According to Lemma 5.1 in [8], it could be demonstrated that the maximum *APNs* for the triangular, regular, square grid, and hexagon-based deployment, denoted by $\gamma_{\text{max}}^{T},\gamma_{\text{max}}^{S},\gamma_{\text{max}}^{H}$, respectively, are expressed as follows [8]:

(8) $$\left\{ \begin{array}{l} \gamma_{\text{max}}^{T}=\frac{3}{2}\sqrt{3}{\left(\text{min}\{r_{s},\frac{\sqrt{3}}{3}r_{c}\}\right)}^{2}\\ \gamma_{\text{max}}^{S}=2{\left(\text{min}\{r_{s},\frac{\sqrt{2}}{2}r_{c}\}\right)}^{2}\\ \gamma_{\text{max}}^{H}=\frac{3}{4}\sqrt{3}{\left(\text{min}\{r_{s},r_{c}\}\right)}^{2} \end{array}\right.$$

For different values of *r_c_*/*r_s_*, we could rewrite the expression of *APNs* as follows:

(9) $$\left\{ \begin{array}{l} \gamma_{\text{max}}^{T}=\frac{\sqrt{3}}{2}{r_{s}}^{2}{\left(\text{min}\{\sqrt{3},\frac{r_{c}}{r_{s}}\}\right)}^{2}\\ \gamma_{\text{max}}^{S}={r_{s}}^{2}{\left(\text{min}\{\sqrt{2},\frac{r_{c}}{r_{s}}\}\right)}^{2}\\ \gamma_{\text{max}}^{H}=\frac{3\sqrt{3}}{4}{r_{s}}^{2}{\left(\text{min}\{1,\frac{r_{c}}{r_{s}}\}\right)}^{2} \end{array}\right.$$

### 3.2. Lattice Deployment under Information Coverage

We now present analysis on the coverage and connectivity on regular pattern-based deployment strategy for lattice WSNs, which are all based on the information coverage model in the above section.

**Lemma 1.**  *For the pattern-based lattice WSNs resulting from deterministic sensor deployment as in Figure 1, the worst (K,ε)-covered points in equilateral triangular, square, and hexagonal grids are centroid of corresponding grids*.

**Figure 3:**
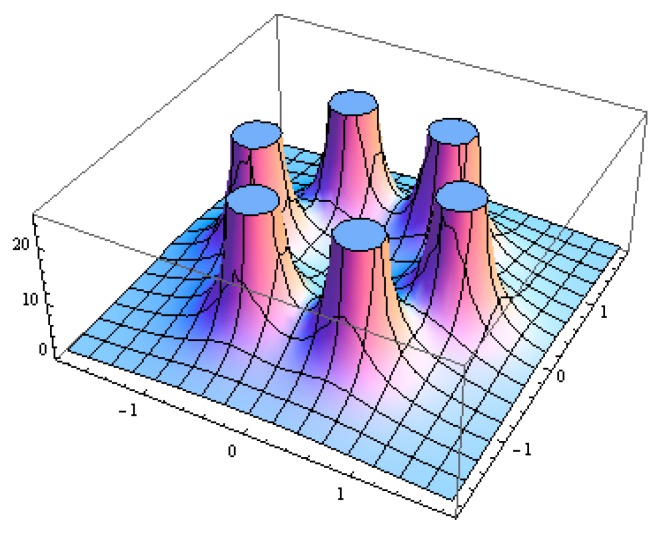
The sensing capacity of six sensors that use the (6,*ε*) coverage model that deployed at vertices of hexagon. The worst-covered point by these three sensors is at the center of the hexagon.

**Proof.**  Here, we will show that the situation under the information coverage model defined in Equation (4), where is the worst (*K,ε*)-covered point by *K* sensors located at vertices of the equilateral triangular, square, and hexagonal grids, and then we have:

(10) $$\underset{\begin{array}{l} d_{i}\in D\\ K=3,4,6 \end{array}}{\text{arg}\;\text{min}}(\text{Pr}\left\{\left|{\tilde{\theta }}_{K}\right|\leq A\right\})=\underset{\begin{array}{l} d_{i}\in D\\ K=3,4,6 \end{array}}{\text{arg}\;\text{min}}(1-2Q(\frac{A}{\sigma \sqrt{{\left(\sum_{i=1}^{K}d_{i}^{-2\alpha }\right)}^{-1}}}))$$

where *d_i_* ∈ **D** implies that it is the distance from interior point to vertices, since the monotonicity of Q-function, then we establish a coordinate system in which the centroid of the equilateral triangular, square, and hexagonal grids is origin (0,0), thus Equation (10) could be converted into:

(11) $$\begin{array}{lll} \text{minimize} & & f(x,y)=\sum_{i=1}^{K}{\left({\left(x-x_{i}\right)}^{2}+{\left(y-y_{i}\right)}^{2}\right)}^{-\alpha }\\ \text{subject to} & & (x,y)\in P,K=3,4,6 \end{array}$$

where (*x_i_,y_i_*) is the coordinate of vertices *i*. and (*x,y*) ∈ *P* denotes that (*x,y*) is the interior point of regular polygon *P*. Since the extremum of continuous function *f*(*x,y*) appear at either stationary points or boundaries, taking some mathematical manipulation and using the same method described in the Appendix of [25], we could find that minimum of *f*(*x,y*) is in the centroid of regular polygon, which can be illustrated in Figure 3 as an example for hexagonal deployment. □

According to Lemma 1, since the worst (*K,ε*)-covered points in equilateral triangular, square and hexagonal grids are centroid of corresponding grids, the maximal normalized distance between vertex and centroid of these deployment patterns can be deduced:

(12) $$\text{Pr}\left\{\left|{\tilde{\theta }}_{K}\right|\leq A\right\})=1-2Q(\frac{A}{\sigma \sqrt{{\left(K\cdot d_{\text{max}}^{-2\alpha }\right)}^{-1}}})\geq \varepsilon$$

when *A* = *σ* and *d*_max_ is the normalized maximum distance, the ${\hat{r}}_{s}$ is the sensing range for (1,*ε*)-covered, there is:

(13) $$\begin{array}{cc} d_{\text{max}}={\left(\frac{\sqrt{K}}{Q^{-1}(\frac{1-\varepsilon }{2})}\right)}^{\frac{1}{\alpha }}, & K=3,4,6 \end{array}$$

**Figure 4:**
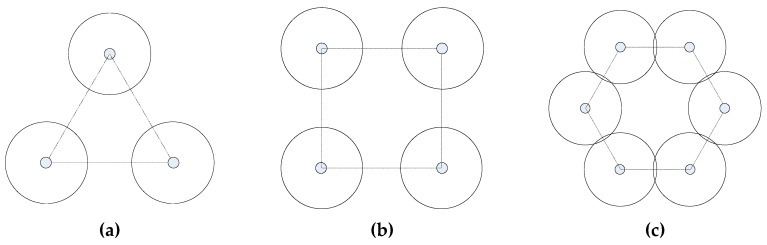
Three common regular patterns of deployment—(**a**) triangle (3,*ε*); (**b**) square (4,*ε*); (**c**) hexagon (6,*ε*), that are under information coverage model.

As a result, maximal actual distance $D_{\text{max}}=\sqrt{K}{\hat{r}}_{s}=\frac{\sqrt{K}r_{s}}{Q^{-1}\left(\frac{1-\varepsilon }{2}\right)},$ this can be used for computation of *γ*_cov_. When *ε* = 0,683 and *α* = 1, then $D_{\text{max}}=\sqrt{K}r_{s},\;K=3,4,6$ can be used for a square, triangle, or hexagon of pattern-based lattice WSNs as in Figure 4, based on Equations (8) and (13), and geometrical computation on *γ*_con_ which is similar in [1]. 

The worst (*K,ε*)-covered points could be utilized to determine the restriction on spacing of the sensor, which is adopted in the deployment under the information coverage model. On the other hand, two sensors are said to be neighbors of each other if their Euclidean distance is at most the communication range *r_c_*, according to the disk communication model, as this is also the fundamental limit for connectivity of WSN. Consequently, the restriction of lattice deployment and relevant *APN* would be redefined.

Similar to the above section, it could be demonstrated that the maximum *APNs* for the triangular, regular, square grid, and hexagon deployment, based on information coverage, denoted by ${\tilde{y}}_{\text{max}}^{T},{\tilde{y}}_{\text{max}}^{S},{\tilde{y}}_{\text{max}}^{H}$, respectively, are expressed as follows:

(14) $$\left\{ \begin{array}{ll} {\tilde{\gamma }}_{\text{max}}^{T}=\frac{\sqrt{3}}{2}{\left(\text{min}\{\frac{3r_{s}}{Q^{-1}(\frac{1-\varepsilon }{2})},r_{c}\}\right)}^{2}, & \text{Triangular}\;(3,\varepsilon )\\ {\tilde{\gamma }}_{\text{max}}^{S}={\left(\text{min}\{\frac{2\sqrt{2}r_{s}}{Q^{-1}(\frac{1-\varepsilon }{2})},r_{c}\}\right)}^{2}, & \text{Square}\;(4,\varepsilon )\\ {\tilde{\gamma }}_{\text{max}}^{H}=\frac{3\sqrt{3}}{4}{\left(\text{min}\{\frac{\sqrt{6}r_{s}}{Q^{-1}(\frac{1-\varepsilon }{2})},r_{c}\}\right)}^{2}, & \text{Hexagonal}\;(6,\varepsilon ) \end{array}\right.$$

When *ε* = 0,683, for different values of *r_c_*_/_*r_s_,* we could rewrite the expression of *APNs* as follows:

(15) $$\left\{ \begin{array}{ll} {\tilde{\gamma }}_{\text{max}}^{T}=\frac{\sqrt{3}}{2}{r_{s}}^{2}{\left(\text{min}\{3,\frac{r_{c}}{r_{s}}\}\right)}^{2}, & \text{Triangular}\;(3,\varepsilon )\\ {\tilde{\gamma }}_{\text{max}}^{S}={r_{s}}^{2}{\left(\text{min}\{2\sqrt{2},\frac{r_{c}}{r_{s}}\}\right)}^{2}, & \text{Square}\;(4,\varepsilon )\\ {\tilde{\gamma }}_{\text{max}}^{H}=\frac{3\sqrt{3}}{4}{r_{s}}^{2}{\left(\text{min}\{\sqrt{6},\frac{r_{c}}{r_{s}}\}\right)}^{2}, & \text{Hexagonal}\;(6,\varepsilon ) \end{array}\right.$$

We have the following analysis results on sensor density requirement for full (*K,ε*)-coverage and full connectivity.

(16) $$\rho_{\text{cov},con}=\left\{ \begin{array}{ll} \frac{1}{\frac{\sqrt{3}}{2}{r_{s}}^{2}{\left(\text{min}\{3,\frac{r_{c}}{r_{s}}\}\right)}^{2}}, & \text{Triangular}\;(3,\varepsilon )\\ \frac{1}{{r_{s}}^{2}{\left(\text{min}\{2\sqrt{2},\frac{r_{c}}{r_{s}}\}\right)}^{2}}, & \text{Square}\;(4,\varepsilon )\\ \frac{1}{\frac{3\sqrt{3}}{4}{r_{s}}^{2}{\left(\text{min}\{\sqrt{6},\frac{r_{c}}{r_{s}}\}\right)}^{2}}, & \text{Hexagonal}\;(6,\varepsilon ) \end{array}\right.$$

where *r_c_* is communication range, for a triangular, a square, and a hexagonal pattern-based WSN for (3,*ε*), (4,*ε*) and (6,*ε*) information coverage respectively. Obviously *r_c_*/*r_s_* could affect the area of the unit Voronoi polygon for each sensor and the deployment performance. 

### 3.3. Dual-Triangular Pattern Deployment

**Figure 5:**
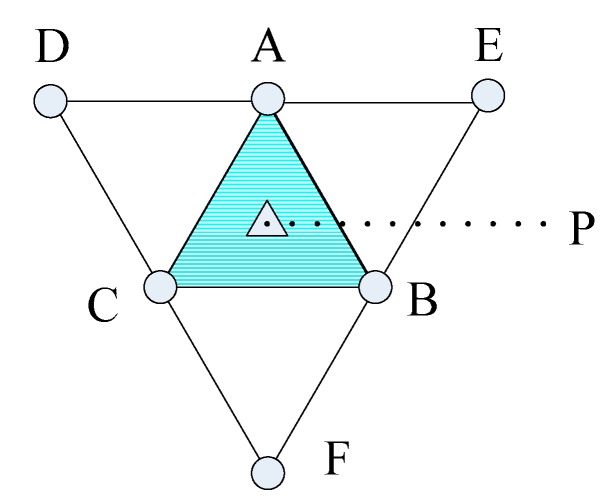
(6,*ε*)-Information coverage for dual-triangular pattern deployment.

As stated in Figure 5, we propose a dual triangular pattern deployment based on novel data fusion strategy. Although it seems the sensor deployment is similar to the deployment as in Figure 4a, its data fusion strategy is much different from it. For example, in original triangular pattern, the points in Δ*ABC* are covered by the sensors *A, B* and *C* on the vertices, so they are (3,*ε*)-covered. Whereas, under new data fusion strategy, the points in Δ*ABC* are not only covered by the contribution of sensors *A, B* and *C* on the vertices of inner triangle, but also covered by the contribution of sensors *D*, *E* and *F* on the vertices of outer triangle, so they are (6,*ε*)-covered, i.e. it takes advantage of data fusion by sensors on vertices of inner and outer triangle grid to achieve both full information coverage and full connectivity.

Based on the similar principle as Lemma 1 since the worst (*K*,*ε*)-covered point in Δ*ABC*, for both inner triangle composed by sensors *A*, *B* and *C* and the outer triangle composed by sensors *D*, *E* and *F*, is the centroid of Δ*ABC*, the maximal distance between vertex *A*, *B* and *C* and centroid *P* of this deployment patterns can be deduced:

(17) $$\text{Pr}\{\left|{\tilde{\theta }}_{K}\right|\leq A\})=1-2Q(\frac{A}{\sigma \sqrt{{\left(3\cdot d_{\text{max}}^{-2\alpha }+3\cdot {\left(2d_{\text{max}}\right)}^{-2\alpha }\right)}^{-1}}})\geq \varepsilon$$

where *d*_max_ is maximal normalized distance between vertex and centroid, when *A* = *σ* and the ${\hat{r}}_{s}$ is the sensing range for (1,*ε*)-covered, there is:

(18) $$d_{\text{max}}=(\frac{\sqrt{\frac{3\cdot (1+2^{2\alpha })}{2^{2\alpha }}}}{Q^{-1}(\frac{1-\varepsilon }{2})}{)}^{\frac{1}{\alpha }}$$

When *ε* = 0,683 and *α* = 1, then the maximal distance *D*_max_ = $\frac{\sqrt{15}}{2}{\hat{r}}_{s}=\frac{\sqrt{15}}{2}r_{s}/Q^{-1}\left(\frac{1-\varepsilon }{2}\right)$, then we have the following analysis result on sensor density requirement for full (6,*ε*) coverage and full connectivity, then it could be demonstrated that the maximum *APNs* for dual-triangular deployment, based on information coverage, denoted by ${\tilde{\gamma }}_{\text{max}}^{DT}$ are expressed as follows:

(19) $$\begin{array}{cc} {\tilde{\gamma }}_{\text{max}}^{DT}=\frac{\sqrt{3}}{2}\left(\text{min}\{\frac{\frac{3\sqrt{5}}{2}r_{s}}{Q^{-1}(\frac{1-\varepsilon }{2})},r_{c}\right\}{)}^{2}, & \text{Dual-Triangular}\;(6,\varepsilon ) \end{array}$$

When *ε* = 0.683 and *α* = 1, for different values of *r_c_*/*r_s_,* we could rewrite the expression of *APNs* as follows:

(20) $$\begin{array}{cc} {\tilde{\gamma }}_{\text{max}}^{DT}=\frac{\sqrt{3}}{2}{r_{s}}^{2}{\left(\text{min}\{\frac{3\sqrt{5}}{2},\frac{r_{c}}{r_{s}}\}\right)}^{2}, & \text{Dual-Triangular}\;(6,\varepsilon ) \end{array}$$

Through some geometrical computation:

(21) $$\begin{array}{cc} \rho_{\text{cov},con}=\frac{1}{\frac{\sqrt{3}}{2}{r_{s}}^{2}{\left(\text{min}\{\frac{3\sqrt{5}}{2},\frac{r_{c}}{r_{s}}\}\right)}^{2}}, & \text{Dual-Triangular}\;(6,\varepsilon ) \end{array}$$

Equation (21) shows that *r_c_*/*r_s_* could determine the optimal deployment patterns for the sensor density based on (*K*,*ε*) coverage.

## 4. Strip-Based Deployment

In this section, we also extend the strip-based deployment to a new pattern, from physical coverage to information coverage, and give some results of its characteristics.

### 4.1. Strip-Based Deployment under Physical Coverage

**Figure 6:**
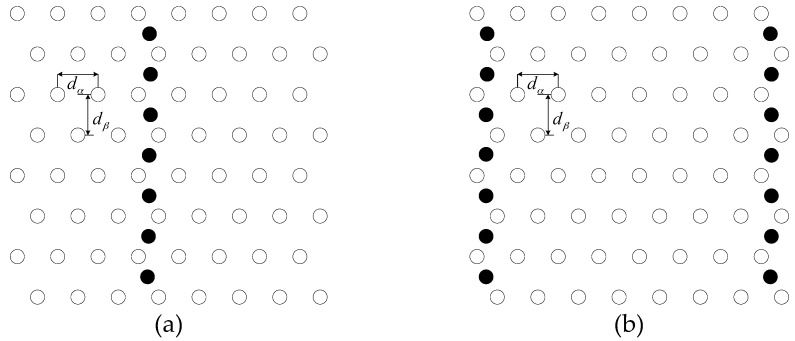
Strip-based deployment for obtaining full coverage with (**a**) 1-connectivity and 2-connectivity (**b**), when *r_c_*/*r_s_* < $\sqrt{3}$. The white-filled dots show the sensor positions that form (**a**) one horizontal strip or (**b**) two horizontal strips. Here, $d_{\alpha }=\text{min}\left\{r_{c},\sqrt{3}r_{s}\right\}$ and $d_{\beta }=r_{s}+\sqrt{r_{s}^{2}-d_{\alpha }^{2}/4}$. The vertical strip of sensor nodes could be removed when *r_c_*/*r_s_* < $\sqrt{3}$ [8].

The strip-based deployment for WSNs was proposed to obtain both full coverage and 1-connectivity or 2-connectivity in Reference [8] (see Figure 6).

Consider an Euclidean plane as the deployment region. A horizontal strip of sensor nodes is composed by placing sensor nodes together with a regular separation of $d_{\alpha }=\text{min}\left\{r_{c},\sqrt{3}r_{s}\right\}$. These strips are composed of horizontally deployed sensor nodes, with alternate rows shifted to the right or left by a distance of $d_{\alpha }/2$. The vertical separation between two adjacent strips is $d_{\beta }=r_{s}+\sqrt{r_{s}^{2}-d_{\alpha }^{2}/4}$. It is noteworthy that while *r_c_*/*r_s_* < $\sqrt{3}$, the adjacent horizontal strip of sensors are not connected, we will give the brief proof on it later. On this occasion, we provide additional sensor nodes at the middle (for 1-connectivity) or left and right boundaries (for 2-connectivity) of the deployment region (the dark-filled dots in Figure 5).

Denote the distance between the sensor nodes of two adjacent horizontal strips by *δ,* which is $\delta =\sqrt{{\left(\frac{d_{\alpha }}{2}\right)}^{2}+d_{\beta }^{2}}$. Thus, we need *N_ad_* sensor nodes to connect a pair of adjacent horizontal strips in order to guarantee 1-connectivity or 2-connectivity, while *N_ad_* can be computed as [8]:

(22) $$N_{ad}=\left\{ \begin{array}{ll} \left\lceil \delta /r_{c}-1\right\rceil & 1-\text{connectivity}\\ 2(\left\lceil \delta /r_{c}-1\right\rceil ) & 2-\text{connectivity} \end{array}\right.$$

**Figure 7:**
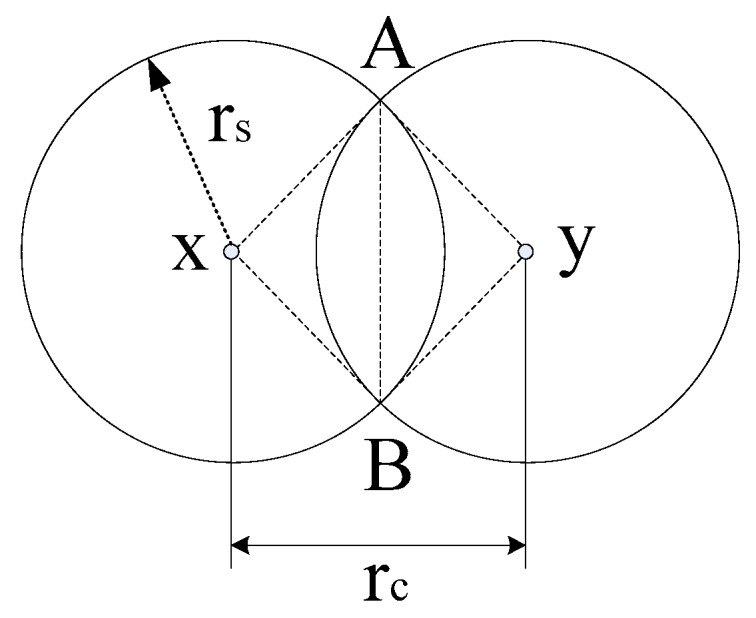
The sensors located at positions *x* and *y* are connected with the feature that *d*(*x,y*) = *r_c_.* Chord *AB* is the connection chord. ∠AxB and ∠AyB are the connection angles.

Then, we provide some definitions about the strip-based deployment which is as same as in [8]:

**Definition 2 [Connection Chord, Connection Angles].**  *Consider two connected neighboring sensor nodes, that are placed at x and y. Thus, there is a common chord between the disks of their sensing coverage, which are* $D_{r_{s}}\left(x\right)$ *and* $D_{r_{s}}\left(y\right)$*, that is illustrated by line segment AB in Figure 7. As the positions (x and y) of these two sensor nodes are varied, the length of their common chord varies synchronously. Given the specified values of r_s_ and r_c_, the shortest possible common chord is addressed as connection chord, and this minimum length is denoted by l(r_s_,r_c_). The central angles related to this shortest common chord, at* $D_{r_{s}}\left(x\right)\;$ *and* $D_{r_{s}}\left(y\right)$ *are called connection angles. We denote these by ϕ(r_s_,r_c_). Notice that l(r_s_,r_c_) = 0 when r_c_ ≥ 2r_s_*.

Let *N*(*r_s_*,*r_c_*) be the minimum number of sensing disks needed for coverage in the strip based deployment. We divide the total number *N*(*r_s_*,*r_c_*) into two parts—*N_h_*(*r_s_*,*r_c_*) that represents the number of sensing disks needed in all horizontal strips (see Figure 6), and *N_v_*(*r_s_*,*r_c_*) that represents the number of sensing disks needed in vertical strips, which could be zero in some condition as in *Lemma 2*. Finally *N*(*r_s_*,*r_c_*) could be expressed as:

(23) $$N(r_{s},r_{c})=N_{h}(r_{s},r_{c})+N_{v}(r_{s},r_{c})$$

**Lemma 2.**  *If r_c_/r_s_ ≥* $\sqrt{3}$*, the adjacent horizontal strip of sensors are natively connected in this case, without any additional sensor nodes needed in vertical strips. i.e., N_v_(r_s_,r_c_) = 0*.

**Proof.**  When *r_c_*/*r_s_* ≥ $\sqrt{3}$, there is $d_{\alpha }=\text{min}\left\{r_{c},\sqrt{3}r_{s}\right\}=\sqrt{3}r_{s}\leq r_{c}$, then the vertical separation between two adjacent strips is $d_{\beta }=r_{s}+\sqrt{r_{s}^{2}-d_{\alpha }^{2}/4}=r_{s}+\sqrt{r_{s}^{2}-{\left(\sqrt{3}r_{s}\right)}^{2}/4}=3r_{s}/2$. Therefore, the distance between two sensors in adjacent strips is $d_{s}=\sqrt{{\left(\frac{d_{\alpha }}{2}\right)}^{2}+d_{\beta }^{2}}$, and the ratio of *d_s_/r_c_* is:

(24) $$\frac{d_{s}}{r_{c}}=\frac{\sqrt{{\left(\frac{d_{\alpha }}{2}\right)}^{2}+{d_{\beta }}^{2}}}{r_{c}}=\frac{\sqrt{{\left(\frac{\sqrt{3}r_{s}}{2}\right)}^{2}+{\left(\frac{3}{2}r_{s}\right)}^{2}}}{r_{c}}=\frac{\sqrt{3}r_{s}}{r_{c}}\leq 1$$

As *d_s_* ≤ *r_c_*, the sensors in adjacent horizontal strip are natively connected, i.e., *N_v_*(*r_s_,r_c_*) = 0. Otherwise, *N_v_*(*r_s_,r_c_*) = *N_ad_*. □

**Figure 8:**
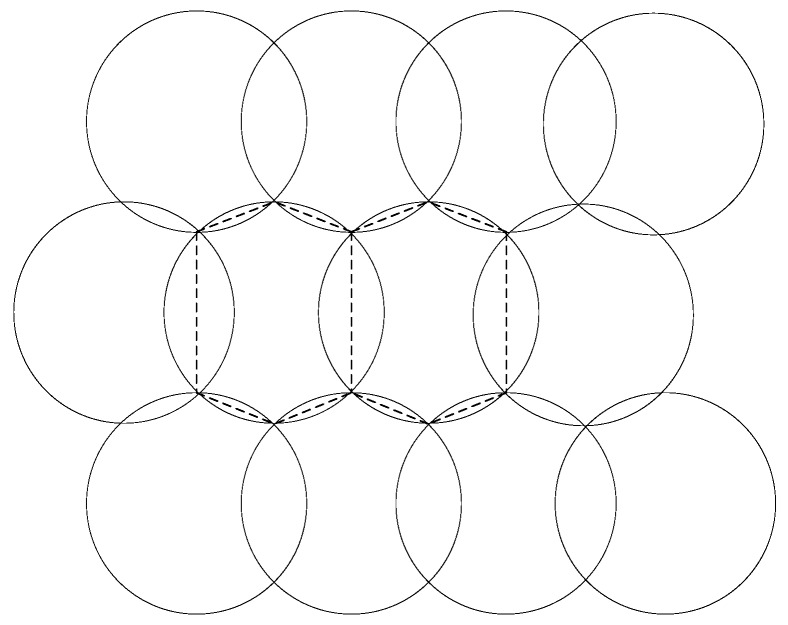
Two of the non-overlapping hexagons that cover the two-dimensional plane, the Voronoi polygon is the polygon formed by the dashed line.

To derive the expression of *N_h_*(*r_s_,r_c_*), we can observe that the two-dimension plane could be tiled with a non-overlapping Voronoi polygons such as the hexagons marked with dotted lines in. Figure 8. It could be found that the area of such hexagon is *APN* for this deployment (in Definition 1).

Therefore, we could use a similar analytical approach as in Section 3, while the maximum *APN* for this deployment is determined by:

(25) $$\gamma_{\text{max}}=n[\frac{1}{2}{r_{s}}^{2}\sin(\varphi )]+(k-n)\frac{1}{2}{r_{s}}^{2}\sin(\frac{2\pi -n\varphi }{k-n})$$

where $\varphi =2arccos\left(\frac{d_{\alpha }}{2r_{s}}\right)$ (the derivation can be found in Appendix A in [8]), the hexagon is divided by some triangles, and all the area of these triangles can be obtained by the sine theorem. In this case, *k* = 6 and *n* = 2, so *k* − *n* = 4, thus we have:

(26) $$\gamma_{\text{max}}={r_{s}}^{2}(\sin(\varphi )+2\sin(\frac{\pi -\varphi }{2}))$$

Then we have the following results on sensor density requirement for strip based deployment to achieve full coverage and connectivity:

(27) $$\rho_{\text{cov},con}=\frac{1}{\gamma_{\text{max}}}=\frac{1}{{r_{s}}^{2}(\sin(\varphi )+2\sin(\frac{\pi -\varphi }{2}))}$$

While *N_A_* is the area of the whole region for deployment, ignoring the boundary effect, thus we could acquire *N_h_*(*r_s_,r_c_*) which is the number of sensors needed in all horizontal strips:

(28) $$N_{h}(r_{s},r_{c})=N_{A}\rho_{\text{cov},con}=\frac{N_{A}}{{r_{s}}^{2}(\sin(\varphi )+2\sin(\frac{\pi -\varphi }{2}))}$$

### 4.2. Strip-Based Deployment Under Information Coverage

As same as in Section 3.2, the restriction of strip-based deployment and relevant APN would be redefined under Information Coverage. We would define a new strip-based deployment based on the (2,*ε*) coverage, in which each point in the region is covered by two sensors cooperatively. This can be denoted as follows:

(29) $$\text{Pr}\{\left|{\tilde{\theta }}_{K}\right|\leq A\})=1-2Q(\frac{A}{\sigma \sqrt{\frac{1}{d_{1}^{-2\alpha }+d_{2}^{-2\alpha }}}})\geq \varepsilon$$

Without loss of generality, we set *A* = *σ* and *α* = 1, while there is *d*_1_ = *d*_2_ = *d*, then we could work out the maximum distance between the center of the sensing disk and the points in the midline of two sensors, while (2,*ε*) coverage could be achieved in this position. This maximal normalized distance ${\hat{d}}_{\text{max}}$ could be computed as follows: when the normalized distance exceeds ${\hat{d}}_{\text{max}},\left(2,\varepsilon \right)$ coverage could not be achieved by the information fusion of two neighboring sensors:

(30) $${\hat{d}}_{\text{max}}=\frac{\sqrt{2}}{Q^{-1}(\frac{1-\varepsilon }{2})}$$

Based on this result, we could extend the conventional strip-based deployment into a new strip-based deployment, which is under information coverage model. In strip-based deployment with physical coverage, the distance between two neighboring sensors in horizontal strip is $\sqrt{3}r_{s}$ at most, which depends on this relation: $d_{\alpha }=\text{min}\{r_{c},\sqrt{3}r_{s})$. When distance of sensors horizontal strip is $d_{\alpha }=\sqrt{3}r_{s},$ then the least overlap of two sensing disks could be achieved, and hereafter the length of common connection chord *l* can be computed as:

(31) $$l=2\sqrt{{r_{s}}^{2}-(\frac{d_{\alpha }}{2}{)}^{2}}=2\sqrt{{r_{s}}^{2}-(\frac{\sqrt{3}r_{s}}{2}{)}^{2}}=r_{s}$$

**Definition 3 [Generalized Connection Chord].**  *Adapting information coverage model, we could define new concept of connection chord, Generalized Connection Chord, which is line segment in the mid-line of two sensors, when the distance between two end points of this line segment is equal to* ${\hat{r}}_{s}$.

Based on this new concept of connection chord, we could compute the horizontal and vertical separation ${\hat{d}}_{\alpha }$ and ${\hat{d}}_{\beta }$, that are under (2,*ε*) coverage.

**Figure 9:**
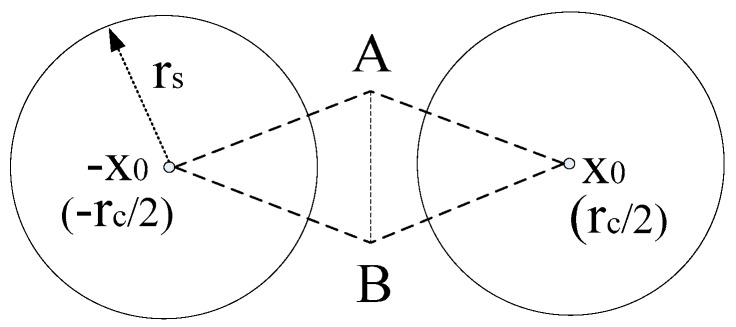
The sensors located at positions *x* and –*x* are connected. Generalized Connection Chord AB is the connection chord with the feature that *d*(*x*_0_,*B*) = *d*(*x*_0_,*A*) ≤ ${\hat{D}}_{max}$, and *l*(*A,B*) = ${\hat{r}}_{s}$.

As in Figure 9, we set the positions of two sensors in horizontal strip as (–*x*_0_,0) and (*x*_0_,0), where *x*_0_ > 0 and the length of generalized connection chord AB is equal to ${\hat{r}}_{s}$. In this case *x*_0_ could be the candidate *x*-coordinate of the sensors’ position which may stretch as far as they could, although the final *x*-coordinate may be determined also by *r_c_* i.e. the sensors would locate at either (±*x*_0_,0) or (±*r_c_*/2,0). We also set positions of end points of generalized connection chord as (0,*y*_0_) and (0,–*y*_0_), where *y*_0_ > 0. Then the maximal distance between the centre of sensing disk and end points of generalized connection chord is ${\hat{D}}_{\text{max}}$ and after removing normalization we have ${\hat{D}}_{\text{max}}={\hat{d}}_{\text{max}}{\hat{r}}_{s}$. Here ${\hat{d}}_{\alpha }=2x_{0}$, and $y_{0}=\sqrt{{\hat{D}}_{\text{max}}^{2}-{\left(\frac{{\hat{d}}_{\alpha }}{2}\right)}^{2}}=\sqrt{{\hat{D}}_{\text{max}}^{2}-x_{0}^{2}}$, then $x_{0}=\sqrt{{\hat{D}}_{\text{max}}^{2}-y_{0}^{2}}$. Since the length of generalized connection chord $l={\hat{r}}_{s}$, there are $y_{0}=\frac{l}{2}=\frac{{\hat{r}}_{s}}{2}$ and following results:

(32) $$x_{0}=\sqrt{{\hat{D}}_{\text{max}}^{2}-{y_{0}}^{2}}=\sqrt{(\frac{\sqrt{2}r_{s}}{Q^{-1}(\frac{1-\varepsilon }{2})}{)}^{2}-{\left(\frac{r_{s}}{2Q^{-1}(\frac{1-\varepsilon }{2})}\right)}^{2}}=\frac{\sqrt{7}r_{s}}{2Q^{-1}(\frac{1-\varepsilon }{2})}$$

(33) $${\hat{d}}_{\alpha }=\text{min}\{r_{c},2x_{0}\}=\text{min}\{r_{c},\frac{\sqrt{7}r_{s}}{Q^{-1}(\frac{1-\varepsilon }{2})}\}$$

(34) $${\hat{d}}_{\beta }=\frac{l}{2}+{\hat{r}}_{s}=\frac{3{\hat{r}}_{s}}{2}=\frac{3r_{s}}{2Q^{-1}(\frac{1-\varepsilon }{2})}$$

the distance between two sensors in adjacent strips under (2,*ε*) coverage is ${\hat{d}}_{s}$:

(35) $${\hat{d}}_{s}=\sqrt{(\frac{{\hat{d}}_{\alpha }}{2}{)}^{2}+{{\hat{d}}_{\beta }}^{2}}=\sqrt{(\frac{{\hat{d}}_{\alpha }}{2}{)}^{2}+(\frac{3r_{s}}{2Q^{-1}(\frac{1-\varepsilon }{2})}{)}^{2}}$$

Adapting (2,*ε*) coverage into strip-based deployment, while we set *ε* = 0.683, then:

(36) $${\hat{d}}_{\alpha }=\text{min}\{r_{c},2x_{0}\}=\text{min}\{r_{c},\sqrt{7}r_{s}\}$$

(37) $${\hat{d}}_{\beta }=\frac{l}{2}+{\hat{r}}_{s}=\frac{3r_{s}}{2}$$

To derive the expression of ${\hat{N}}_{h}\left(r_{s},r_{c}\right)$, the number of sensors needed in all horizontal strips for (2,*ε*) coverage, we could also find that the area of such hexagon is *APN* for this deployment. Therefore, we could use similar way as in physical coverage, while the maximum *APN* for this deployment is determined by:

(38) $${\hat{\gamma }}_{\text{max}}={{\hat{d}}_{s}}^{2}\sin(\hat{\varphi })+2{\hat{d}}_{s}{\hat{r}}_{s}\sin(\frac{\pi -\hat{\varphi }}{2})$$

where $\hat{☐}\hat{\varphi }=2arccos\left(\frac{{\hat{d}}_{\alpha }}{2{\hat{d}}_{s}}\right)$, then we have the following results on sensor density requirement for strip deployment to achieve (2,*ε*) coverage and connectivity:

(39) $${\hat{\rho }}_{\text{cov},con}=\frac{1}{{\hat{\gamma }}_{\text{max}}}=\frac{1}{{{\hat{d}}_{s}}^{2}\sin(\hat{\varphi })+2{\hat{d}}_{s}{\hat{r}}_{s}\sin(\frac{\pi -\hat{\varphi }}{2})}$$

when ${\hat{N}}_{A}$ is the area of the whole region for this deployment to achieve (2,*ε*) coverage and connectivity, ignoring the boundary effect, thus there is:

(40) $${\hat{N}}_{h}(r_{s},r_{c})={\hat{N}}_{A}{\hat{\rho }}_{\text{cov},con}=\frac{N_{A}}{{{\hat{d}}_{s}}^{2}\sin(\hat{\varphi })+2{\hat{d}}_{s}{\hat{r}}_{s}\sin(\frac{\pi -\hat{\varphi }}{2})}$$

since the total number $\hat{N}\left(r_{s},r_{c}\right)$ of sensors needed into two parts: ${\hat{N}}_{h}\left(r_{s},r_{c}\right)$ the number of sensing disks needed in all horizontal strips, and ${\hat{N}}_{v}\left(r_{s},r_{c}\right)$ the number of sensing disks needed in vertical strips, there is:

(41) $$\hat{N}(r_{s},r_{c})={\hat{N}}_{h}(r_{s},r_{c})+{\hat{N}}_{v}(r_{s},r_{c})$$

(42) $${\hat{N}}_{v}(r_{s},r_{c})=\left\{ \begin{array}{lll} N_{ad} & & r_{c}<\frac{\sqrt{7}r_{s}}{Q^{-1}(\frac{1-\varepsilon }{2})}\\ 0 & & r_{c}\geq \frac{\sqrt{7}r_{s}}{Q^{-1}(\frac{1-\varepsilon }{2})} \end{array}\right.$$

We can explain it using the following lemma, as similar as in strip-based deployment with physical coverage.

**Lemma 3.**  *For the (2,ε) coverage model, if* $r_{c}\geq \frac{\sqrt{7}r_{s}}{Q^{-1}\left(\frac{1-\varepsilon }{2}\right)}$*, the adjacent horizontal strip of sensors are natively connected, without any additional sensor nodes needed in vertical strips. i.e.,* ${\hat{N}}_{v}\left(r_{s},r_{c}\right)=0.$

**Proof.**  The distance between two sensors in adjacent strips under (2,ε) coverage is ${\hat{d}}_{s}$, when $r_{c}\geq \frac{\sqrt{7}r_{s}}{Q^{-1}\left(\frac{1-\varepsilon }{2}\right)}$, since ${\hat{d}}_{\alpha }=\text{min}\left\{r_{c},\frac{\sqrt{7}r_{s}}{Q^{-1}\left(\frac{1-\varepsilon }{2}\right)}\right\},$ there is ${\hat{d}}_{\alpha }=\frac{\sqrt{7}r_{s}}{Q^{-1}\left(\frac{1-\varepsilon }{2}\right)}\leq r_{c}$, then we have:

(43) $$\begin{array}{cc} {\hat{d}}_{s} & =\sqrt{(\frac{{\hat{d}}_{\alpha }}{2}{)}^{2}+{{\hat{d}}_{\beta }}^{2}}=\sqrt{(\frac{{\hat{d}}_{\alpha }}{2}{)}^{2}+{\left(\frac{3r_{s}}{2Q^{-1}(\frac{1-\varepsilon }{2})}\right)}^{2}}\\ & =\sqrt{{\left(\frac{\sqrt{7}r_{s}}{2Q^{-1}(\frac{1-\varepsilon }{2})}\right)}^{2}+{\left(\frac{3r_{s}}{2Q^{-1}(\frac{1-\varepsilon }{2})}\right)}^{2}}=\frac{2r_{s}}{Q^{-1}(\frac{1-\varepsilon }{2})} \end{array}$$

Therefore if we compare ${\hat{d}}_{s}$ with *r_c_*, we have:

(44) $$r_{c}\geq \frac{\sqrt{7}r_{s}}{Q^{-1}(\frac{1-\varepsilon }{2})}>\frac{2r_{s}}{Q^{-1}(\frac{1-\varepsilon }{2})}={\hat{d}}_{s}$$

which implies that adjacent horizontal strip of sensors are natively connected in this case, without any additional sensor nodes needed in vertical strips. □

## 5. Discussion and Extension

In this section, we discuss the results we have presented and the try to extend our work to deal with some new issues.

### 5.1. Interrelation Between Coverage and Connectivity

To achieve both full coverage and full connectivity in WSN, every point in the given region should be covered and all sensors in the network should be connected. The analysis of the above sections on coverage and connectivity could be combined. Equations (9), (15), (20), (26) and (38) give the maximal area of the Voronoi polygons for full connectivity and full coverage, respectively, which is included in the function min{ }, for a given sensor in several lattice-based deployments. We could find that the smaller one is the design bottleneck and the ratio of the communication range to the sensing range plays an important role on the area of the Voronoi polygon for each sensor and the deployment efficiency. When *r_c_*/*r_s_* is very small, *r_c_* plays a pivotal role on the deployment scheme. As *r_c_* increases beyond some boundary, it could not improve deployment efficiency any more.

Comparing those equations we could find the information coverage can expand this boundary so that *r_c_* could take effect in a wider range. Otherwise, hexagon, square and triangle deployment could achieve at least 3-connectivity, 4-connectivity, 6-connectivity respectively, while strip-based deployment could achieve at least 1-connectivity and 2-connectivity, in both physical coverage and information coverage model. The one difference is that there is different boundaries of *r_c_*/*r_s_* between physical coverage and information coverage, beyond this boundary, the deployment scheme could achieve more connectivity than it of under the boundary.

### 5.2. Extension to Information N-Coverage

In some applications or for fault tolerance, a point should be covered by more than one sensor node physically, i.e., its distances to N adjacent sensors should all be within the sensing range. This physical N-coverage makes use of redundant sensors to achieve robustness of coverage. When up to *N* − 1 sensors stop working, the point could still be covered physically. In the similar way, robustness of coverage in information coverage could also be achieved by employing redundant sensors. To achieve N robustness, a point should be N-(*K*,*ε*)-covered. This requires that any *K* out of *K* + *N* adjacent sensors of a point could perform (*K*,*ε*)-coverage at the point. In [10], Wang explained this situation, whereas the detailed scheme was not presented. Here, we try to propose a scheme as an instance that could achieve 4-(2,*ε*)-coverage, so as to explore the capability of this extended version of regular deployment in information coverage.

**Figure 10:**
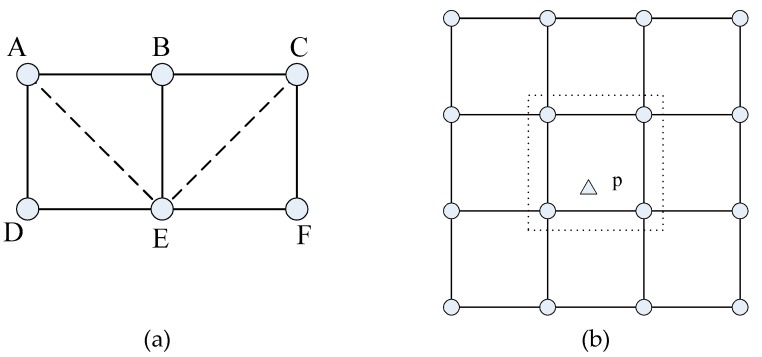
Illustration of 4-(2,*ε*)-coverage: (**a**) data fusion by two sensor (**b**) fault tolerance for 4-(2,*ε*)-coverage.

Figure 10a shows the 4-(2,*ε*)-coverage, sensors A and C carry out data fusion to (2,*ε*)-cover the whole region of ACDF, while point E is the worst (2,*ε*)-covered point in ACDF. This deployment could achieve 4-(2,*ε*)-coverage, since point p could be (2,*ε*)-covered unless more than three adjacent sensors fail, which are sensors indicated in the dashed frame in Figure 10b.

Just like in Equation (30), when the distance exceeds ${\hat{D}}_{\text{max}}$, (2,*ε*)-coverage could not be achieved by the information fusion of two neighboring sensors:

(45) $${\hat{D}}_{\text{max}}=\frac{\sqrt{2}r_{s}}{Q^{-1}(\frac{1-\varepsilon }{2})}$$

Then the length of Voronoi polygons is $l=\text{min}\left(\frac{\sqrt{2}}{2}{\hat{d}}_{\text{max}},r_{c}\right)$, and the *APN* can be expressed as:

(46) $$\gamma_{4,\max}^{S}=\left(\text{min}(\frac{\sqrt{2}}{2}{\hat{d}}_{\text{max}},r_{c}\right){)}^{2}$$

### 5.3. Design Issue for Regular Deployment

The results in the above sections on coverage and connectivity are all based on mathematical analysis. In the practical application, all the issues and factors must be taken in to account.

(1) Number of sensors needed

In different schemes of deployment, this is principal issue we discussed in this paper, since that is the main economic cost of a wireless sensor networks, and it reveals the efficiency of work patterns for WSNs.

(2) Energy cost

We will examine the total energy cost needed for messaging from the event point to the sink in simulations, because this issue is also very important for WSNs for its own characteristics of energy. First, the total energy cost is composed of three parts: (a) the energy cost from sending and relaying messages; (b) the energy cost from receiving messages; (c) the energy cost of sensor collaboration in information coverage, while this part is zero in physical coverage. In information coverage, the hexagon (6,*ε*), square (4,*ε*), triangle (3,*ε*), strip-based (2,*ε*) and dual-triangle (6,*ε*) deployments need to transmit 5, 3, 2, 1 and 5 data packets to perform data fusion between collaborating sensors.

(3) Tolerance

In Section 5.2, we discussed an extension to information N-coverage that is for fault tolerance. Furthermore, there is some other tolerance issues. Since events that happen at a particular position could be estimated with a guaranteed estimation error, we could use the probability that the absolute value of the estimation error is less than a given threshold A, to measure how well a point is covered. However, in practical application, the parameter *σ_i_* of noises cannot be determined accurately, so a usual method is adapting a more relax threshold to guarantee the detection probability for information coverage. We take different threshold A to make the threshold *ε* higher, so that whole coverage has some tolerance. Speaking overall, to develop the regular deterministic deployment in WSNs to achieve both connectivity and coverage, we must consider all the above and some other issues and could use the following steps:

(1) List out all the issues and factors for your WSN applications, and sort them by importance, doing some quantitative descriptions if possible.
(2) Use the results or analysis method proposed in this paper, while taking the issues and factors into account, to design a deployment scheme for this specific WSN application.
(3) Under constraint conditions, optimize the parameters in order to make the whole network as robust as possible.

## 6. Numerical Results

In this section, we compare the numbers of sensors needed to achieve both full coverage and connectivity for all the aforementioned deployments. The whole area for deployment is a two dimension plane which is 1000 m × 1000 m, we set the sensing range *r_s_* = 30 m for physical coverage, and the communication range 20 m ≤ *r_c_* ≤ 120 m, while different deployments are used. For simplicity, the number of sensors needed to cover the boundary of the area is not considered.

### 6.1. Deployment under Physical Coverage

**Figure 11:**
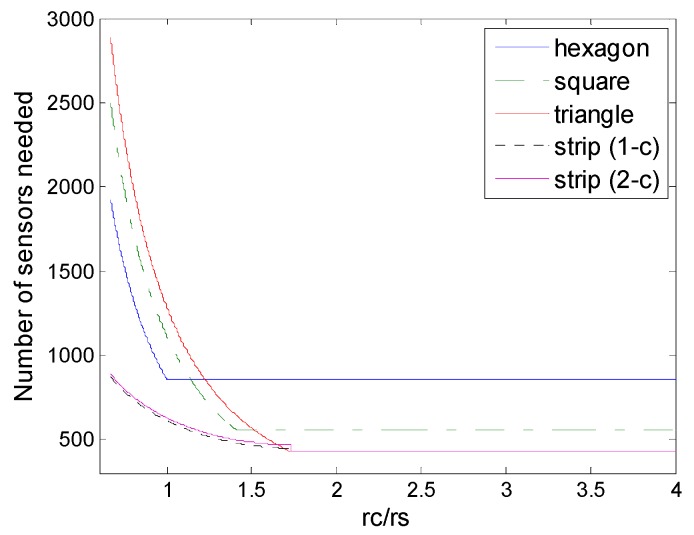
Number of sensors needed in the different schemes of deployment under physical coverage model (hexagon, square, triangle, and strip-based deployment for 1-connectivity and 2-connectivity) to guarantee full coverage and connectivity for various values of *r_c_*/*r_s_*.

Figure 11 shows the comparison of strip-based deployments and regular pattern-based deployments under the physical coverage model. We could find that the strip-based deployments under physical coverage outperform the regular pattern based deployments under physical coverage in terms of the numbers of sensors needed. This means that strip-based deployments could be more suitable under physical coverage conditions, when *r_c_*/*r_s_* is in the low level, there is an obvious advantage.

### 6.2. Strip-Based Patterns

**Figure 12:**
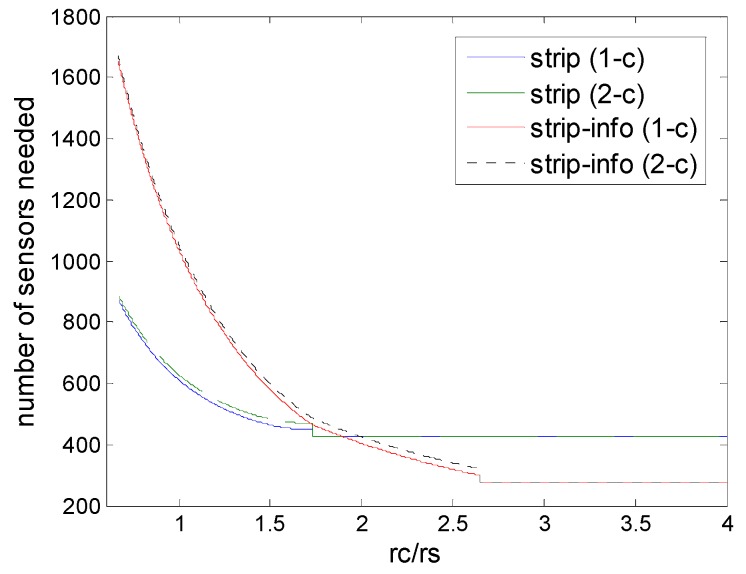
Number of sensors needed in strip-based deployments, that are strip-based deployment under physical coverage model for 1-connectivity and 2-connectivity, and strip-based deployment under (2,*ε*) information coverage for 1-connectivity and 2-connectivity) to guarantee full coverage and connectivity for various values of *r_c_*/*r_s_*.

Figure 12 shows the comparison of strip-based deployments under physical and information coverage model. First, the numbers of sensors needed by the two strip-based deployments (1-connectivity and 2-connectivity) are very close, despite the fact that different coverage models are used; second, we could find that when *r_c_*/*r_s_ ≥* 2, strip-based deployments under information coverage outperform the corresponding deployments under physical coverage in terms of numbers of sensors needed, although it is worse when *r_c_*/*r_s_* is very low. This means that strip-based deployments could be suitable under information coverage, when *r_c_*/*r_s_* is in the high level, since in this case, the ascendency of the information coverage would be adequately exhibited. This is because when the *r_c_*/*r_s_* is relatively high, the bottleneck of the deployment is determined by the sensing range, and collaborative data fusion can be used to improve the performance at this time.

### 6.3. Regular Pattern Based Deployments

**Figure 13:**
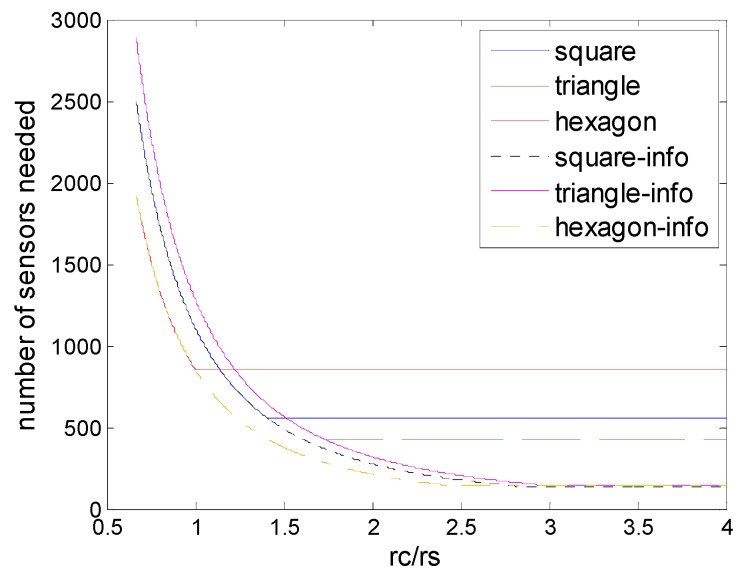
Number of sensors needed in the different schemes of deployment (hexagon, square, triangle, hexagon (6,*ε*), square (4,*ε*), triangle (3,*ε*)) to guarantee full coverage and connectivity for various values of *r_c_*/*r_s_*.

Figure 13 shows the comparison of regular pattern-based deployments under physical and information coverage model. First, for all regular pattern-based deployments, information coverage could be superior to physical coverage currently, in terms of numbers of sensors needed. When *r_c_*/*r_s_* < 1, *r_c_*/*r_s_* < $\sqrt{2}$, *r_c_*/*r_s_* < $\sqrt{3}$ the deployments under information coverage model would be degraded into the corresponding deployments under physical coverage model. That is because when the *r_c_*/*r_s_* level is low, the communication range becomes the main bottleneck of the deployment, while when the *r_c_*/*r_s_* level is relatively high, these deployments could break through the bottleneck of the sensing range so that advantages of information coverage could be reflected, at this time regular pattern based deployments under the information coverage are better than those deployments under physical coverage. Figure 13 shows that *r_c_*/*r_s_* determines the optimal deployment patterns for the sensor density and no pattern is always the best for all the cases. However, it is observed that improvements in sensor density increase as the number of collaboration sensors.

### 6.4. Deployment under Infomation Coverage

**Figure 14:**
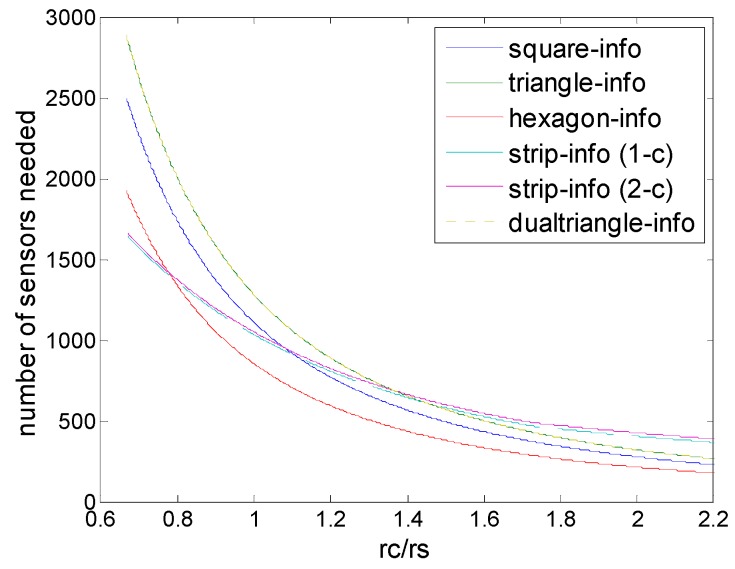
Number of sensors needed in the different schemes of deployment (hexagon (6,*ε*), square (4,*ε*), triangle (3,*ε*) strip-based (2,*ε*) for 1-connectivity and 2-connectivity, dual-triangle (6,*ε*)) to guarantee full coverage and connectivity when *r_c_*/*r_s_* is (0.6, 2.2).

**Figure 15:**
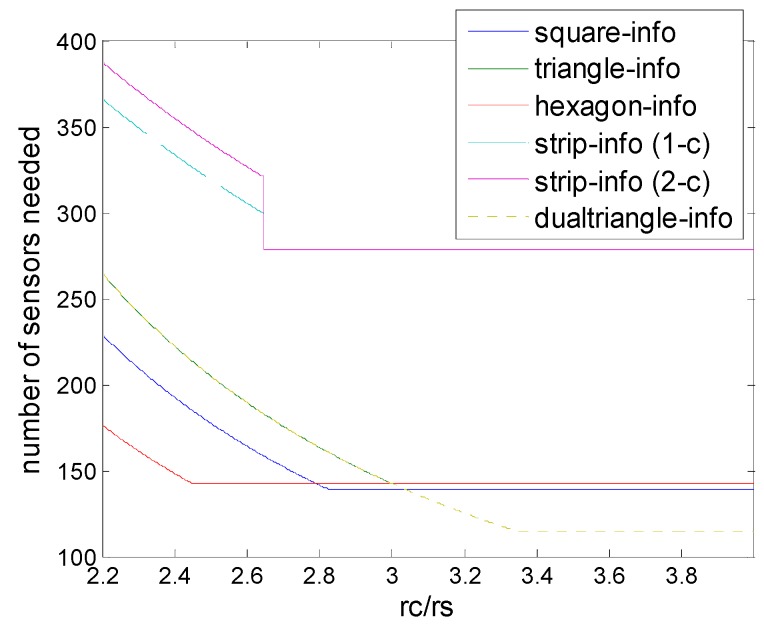
Number of sensors needed in the different schemes of deployment (hexagon (6,*ε*), square (4,*ε*), triangle (3,*ε*), strip-based (2,*ε*) for 1-connectivity and 2-connectivity, dual-triangle (6,*ε*)) to guarantee full coverage and connectivity when *r_c_*/*r_s_* is (2.2, 4).

Figure 14 and Figure 15 show the comparison of all deployments under information coverage model, including the two newly proposed schemes, strip-based information coverage and dual triangular information coverage. For the clarity of illustration, we divided the range of *r_c_*/*r_s_* into two parts, which is low level and high level. As in Figure 14 we can see that for low level of *r_c_*/*r_s_*, hexagonal deployment nearly outperforms all the other deployments under information coverage in general, despite there being a small part of the range, when *r_c_*/*r_s_* < 0.8, that strip-based deployments seems better.

On the other hand, for low level of *r_c_*/*r_s_* hexagonal deployment also has some advantage when *r_c_*/*r_s_* < 2.8, and dual triangular pattern outperforms all the other deployments under information coverage, when *r_c_*/*r_s_* > 3.04, which is shown in Figure 15.

When *r_c_*/*r_s_* ≤ 3 the dual triangular pattern coincides with the equilateral triangle pattern. The dual triangular pattern outperforms the triangular/square/hexagonal patterns under the information coverage model, while the *r_c_*/*r_s_* is higher than 3, 3.04 and 3, respectively. In particular, it is observed that when *r_c_*/*r_s_* increases to a certain high level, the proposed dual triangular pattern deployment could break through the limits on the original triangle, square, and hexagon deployment patterns, and extend the bound on sensor density requirement based on information coverage.

We can see that regardless of hexagon or dual triangular information coverage, due to the fact that the number of sensors is the most in collaborative data fusion feature, it can achieve high efficiency, while the communication consumption is the most. Eventually there are some tradeoffs between the number of sensors and communication overhead, which need to be considered in the specific application.

### 6.5. Deployment under Different Threshold

In real world applications, the standard deviations *σ_i_* of noises could not be determined easily, so we usually adapt a more relaxed threshold to guarantee the detection probability for information coverage. We do some simple simulation about this situation, where we take different thresholds A to make the threshold *ε* higher, the whole coverage has some tolerance.

**Figure 16:**
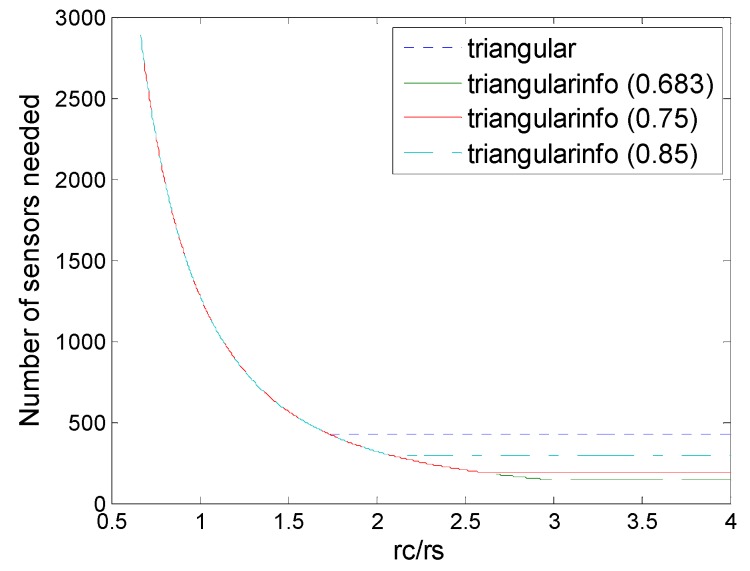
Number of sensors needed in the triangle deployment and triangle (3,*ε*) deployment with various values of threshold *ε* (0.683, 0.75, 0.85).

**Figure 17:**
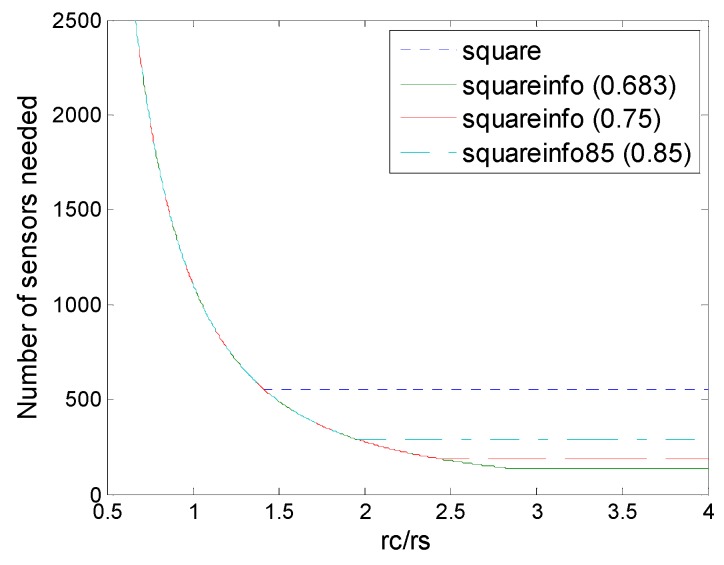
Number of sensors needed in the square deployment and square (4,*ε*) deployment with various values of threshold *ε* (0.683, 0.75, 0.85).

**Figure 18:**
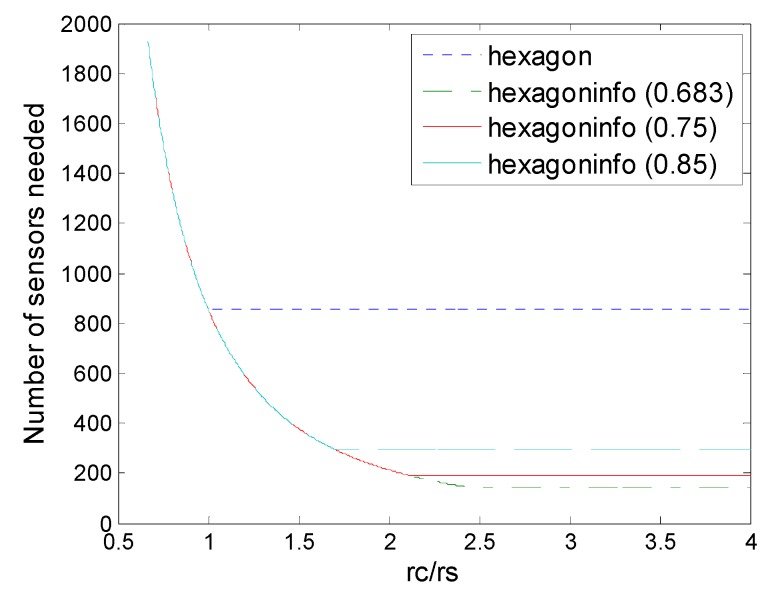
Number of sensors needed in the hexagon deployment and hexagon (6,*ε*) deployment with various values of threshold *ε* (0.683, 0.75, 0.85).

**Figure 19:**
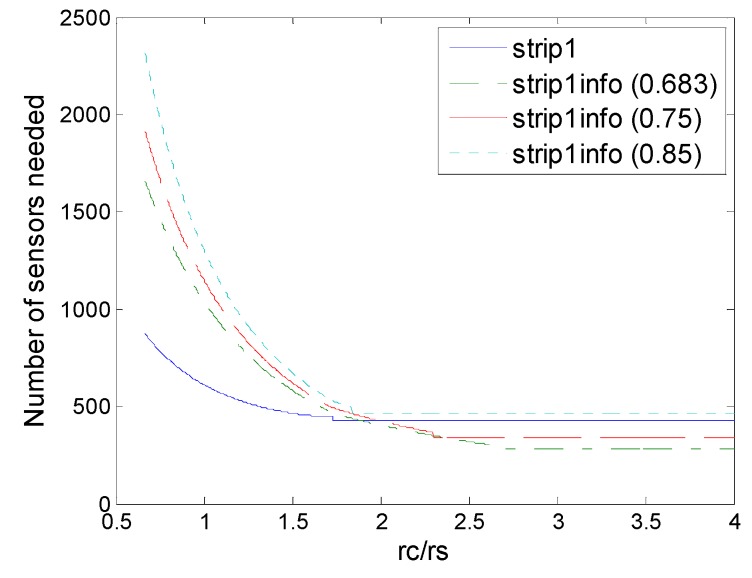
Number of sensors needed in the strip-1 deployment and strip-1 (2,*ε*) deployment with various values of threshold *ε* (0.683, 0.75, 0.85).

**Figure 20:**
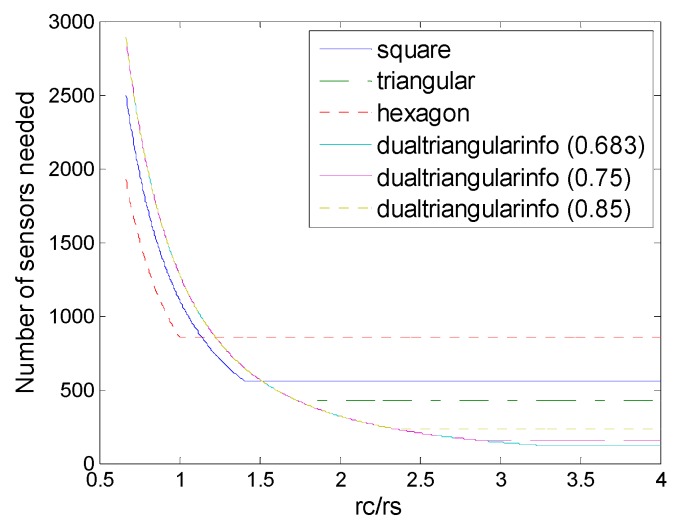
Number of sensors needed in the different schemes of deployment (hexagon, square, triangle, and dual-triangle (6,*ε*) with various values of threshold *ε* (0.683, 0.75, 0.85)).

Figure 16, Figure 17, Figure 18, Figure 19 and Figure 20 show the comparison of all deployments under the information coverage model with various values of threshold *ε*. We can observe that with the threshold *ε* increased for tolerance, the deployment in information coverage becomes more closed compared to that in physical coverage. However, different patterns have different degeneration, so strip-based deployment in information coverage has less advantages, while a dual triangular pattern in information coverage has some superiority.

### 6.6. Energy Cost on Messaging

We examine the total energy cost needed for messaging from the event point to sink. We consider an extreme case that the event occurs at the upper left corner of the two dimension plane, while the sink is at lower right corner. We do the simulation about the total energy cost needed for messaging this from the event point to sink. The energy model and parameters are set as in [35] in Table 2.

**Figure 21:**
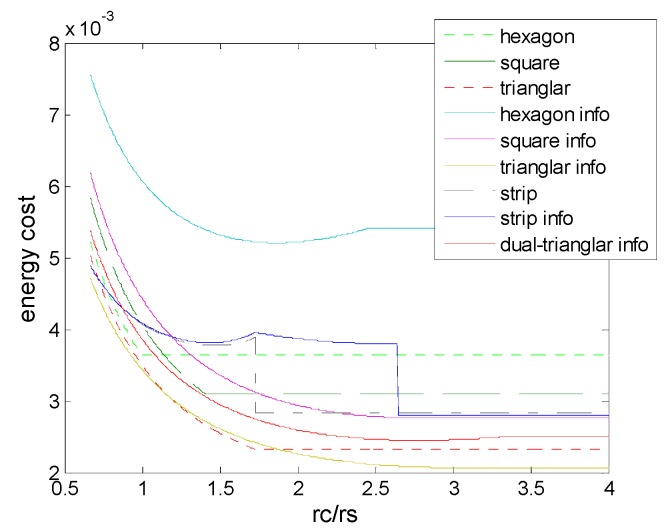
Total energy cost needed for messaging to sink in the different schemes of deployment.

*E_elec_* is the energy dissipated per bit to run the transmitter or the receiver circuit, *ε_fs_* depend on the transmitter amplifier model, *E_DA_* is the processing cost of a bit per report to the sink, and we set a 512_bit message that needs to be sent to sink. Figure 21 shows the comparison of total energy cost for all deployments with various *r_c_/r_s_*, where we could see that the deployment in information coverage does not always have the advantage, due to the impact of the energy cost by sensor collaboration. However, we could find that triangle, triangle (3,*ε*) and dual-triangle (6,*ε*) outperform the other deployment patterns as it’s the nature of the triangle shape, and the triangle (3,*ε*) can combine the advantages of two aspects.

### 6.7. N-Coverage

In Section 5.2, we discuss a little about N-coverage for fault tolerance in, here we do some simulation to validate some discussion and the extension scheme we proposed.

**Figure 22:**
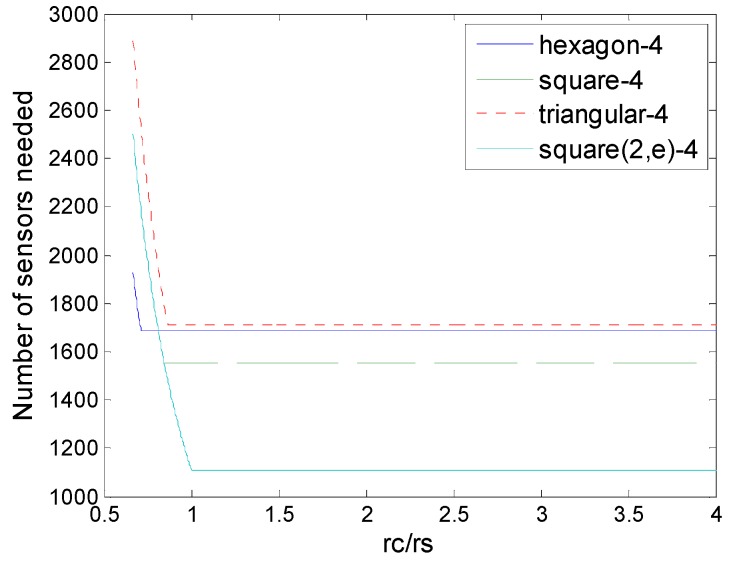
Number of sensors needed in the different schemes of 4-coverage (hexagon, square, triangle, and square (2,*ε*)), square (2,*ε*) is the 4-(2,*ε*)-coverage we proposed in Section 5.2.

Figure 22 show the comparison of number of sensors needed in the different schemes of 4-coverage with various *r_c_/r_s_*, we could find the square 4-coverage in information coverage model ,which is proposed in Section 5.2, has superiority mostly, unless when *r_c_/r_s_* is very small. That superiority comes from the data fusion of sensor collaboration. There is another way to achieve robustness is to mobilize more sensors to recover a given point in the information coverage. For example, if a point could not be (*K,ε*)-covered due to any sensor failure, we could find another two sensors to carry out (*K* + 1*,ε*)-cover the point. Nevertheless, this is a more challenging issue in the mathematical model, so we leave it as an open problem for future research.

## 7. Conclusions

In this paper, some results on optimal regular deployment patterns to achieve information coverage and connectivity as the variety of *r_c_/r_s_* are provided, which are all based on data fusion by sensor collaboration, and a novel data fusion strategy for deployment patterns is proposed. For regular pattern-based lattice WSNs, the relation between variety of *r_c_/r_s_* and density of sensors needed to achieve information coverage and connectivity is deduced in closed form, in order to judge how good are some regular deployment patterns in information coverage, when the value of *r_c_/r_s_* is varied. Then a dual triangular pattern deployment based on novel data fusion strategy is proposed, which could utilize collaborative data fusion more efficiently, while *r_c_/r_s_* is at a high level. The strip-based deployment is also extended to a new pattern to achieve information coverage and connectivity, and the results of its characteristic are deduced in closed form. Finally the efficiencies of all patterns of deployment are analyzed by discussion and compared by some simulations and it is worth to note that the following remarks can be found:

(1) There is very limited advantage for strip-based deployment in information coverage, since its strategy is collaboration by two sensors, which could not benefit from data fusion more deeply.
(2) Regular deployment in information coverage cannot always outperform it in physical coverage, as value of *r_c_/r_s_* is varied. When the *r_c_/r_s_* is at a very low level, deployment in physical coverage could be a good choice instead, since there is extra cost for data fusion in information coverage.

Although these new deployment patterns are based on information coverage model, some other data fusion strategy [32,33,34] could also be adapted to this new kind of deployment, by similar schemes for the future studies.

## Figures and Tables

**Table 1:** Related studies about connectivity and coverage for WSNs.

References	Issue	Coverage Model	*r_c_*/*r_s_*
[1,8]	Connectivity, Coverage	Physical coverage	Variety
[3,5,6,7,26,27,28,29,30,31]	Connectivity, Coverage	Physical coverage	Fixed
[9,10,11,14]	Connectivity, Coverage	Information coverage	Fixed
[12,13]	Connectivity, Coverage	Probabilistic coverage	Fixed
[15]	Connectivity, Barrier Coverage	Information coverage	Fixed
[16]	Coverage	Probabilistic coverage	Fixed
[17]	3D Coverage	Probabilistic coverage	Fixed
[18]	Connectivity, Coverage	Data fusion	Fixed
[2,19,20]	Connectivity, Coverage	Heterogeneous disks	Variety
[21,22,23,24]	Multiple Connectivity, Multiple Coverage	Physical coverage	Fixed
[25]	Connectivity, Coverage	Probabilistic coverage	Fixed
[32,33,34]	Connectivity, Coverage	Data fusion	Fixed

**Table 2:** Radio characteristics used in simulations.

Operation	Energy Dissipated
Transmitter/Receiver Electronics	*E_elec_* = 50 nJ/bit
Data Aggregation	*E_DA_* = 50 nJ/bit/report
Transmit Amplifier	*ε*_fs_ = 50 nJ/bit/report

## Abbreviations

The following abbreviations are used in this manuscript:

WSN	Wireless Sensor Networks
XSM	Extreme Scale Mote
MSE	Mean Squared Error
APN	Area Per Node
